# Clinical characteristics of congenital cervical atresia based on anatomy and ultrasound: a retrospective study of 32 cases

**DOI:** 10.1186/2047-783X-19-10

**Published:** 2014-02-21

**Authors:** Zhihong Xie, Xiaoping Zhang, Jiandong Liu, Ningzhi Zhang, Hong Xiao, Yongying Liu, Liang Li, Xiaoying Liu

**Affiliations:** 1 Department of Obstetrics and Gynecology, Fuyang Clinical Institute, Anhui Medical University, No. 63 Luci Street, Fuyang 236003, China; 2 Department of Pathology, Fuyang Clinical Institute, Anhui Medical University, No. 63 Luci Street, Fuyang 236003, China; 3 Department of Obstetrics and Gynecology, Fuyang Fifth People Hospital, Fuyang 236037, China

**Keywords:** cervicovaginal operation, congenital cervical atresia, Müllerian duct anomaly

## Abstract

**Background:**

To explore the clinical characteristics of congenital cervical atresia.

**Methods:**

This retrospective analysis included 32 cases of congenital cervical atresia treated from March 1984 to September 2010. The anatomic location, ultrasonic features, surgical treatments, and outcomes were recorded.

**Results:**

Based on clinical characteristics observed during preoperative ultrasound and intraoperative exploration, congenital cervical atresia was divided into four types. Type I (*n*?=?22/32, 68.8%) is incomplete cervical atresia. Type II (*n*?=?5/32, 15.6%) defines a short and solid cervix with a round end; the structure lacked uterosacral and cardinal ligament attachments to the lower uterine body. Type III (*n*?=?2/32, 6.3%) is complete cervical atresia, in which the lowest region of the uterus exhibited a long and solid cervix. Type IV (*n*?=?3/32, 9.4%) defines the absence of a uterine isthmus, in which no internal os was detected, and a blind lumen was found under the uterus.

**Conclusions:**

Observations of clinical characteristics of congenital cervical atresia based on the anatomy and ultrasound may inform diagnosis and treatment strategy.

## Background

Congenital cervical atresia is a relatively rare Müllerian duct anomaly of the female reproductive tract that was first reported by Ludwig in 1900. It is associated with acute or chronic abdominal or pelvic pain and reproductive problems [1,2]. The management of women with congenital cervical atresia remains controversial [3].

Congenital cervical atresia must be classified, so that the condition can be considered distinct from other Müllerian duct anomalies in terms of diagnosis and treatment selection. Four systems have been proposed for the classification of congenital cervical atresia [4]: the system of the American Fertility Society (now the American Society of Reproductive Medicine) [5]; the embryological-clinical classification system of genito-urinary malformations [6,7]; the ‘Vagina, Cervix, Uterus, Adnex-associated Malformations’ system, which is based on the ‘tumor nodes metastases’ principle in oncology [8]; and the new European Society of Human Reproduction and Embryology/European Society for Gynaecological Endoscopy classification system developed by the CONUTA working group [9]. These classifications have limitations in terms of effective categorization of anomalies, simplicity, and clinical application [9].

Surgical interventions for congenital cervical atresia range from complete hysterectomy with canalization to conservative options, such as uterine cavity catheterization [10-14]. As a high level of surgical expertise is required to conduct complex reconstructive procedures, many clinicians choose hysterectomy as the optimal primary surgical treatment for the malformation [15].

Most reports on congenital cervical atresia in the literature refer to individual cases. Owing to the clinical importance of congenital cervical atresia, studies including large numbers of patients will be useful to understand the pathogenesis and management of the condition [16]. In this study, we retrospectively studied the clinical characteristics of 32 cases of congenital cervical atresia during preoperative ultrasound and intraoperative exploration. Based on our findings, we identified four types of congenital cervical atresia. Classifying congenital cervical atresia according to anatomy and ultrasound may inform diagnosis and treatment strategy.

## Methods

### Patients

This study was a retrospective analysis of patients who presented with congenital cervical atresia at the Fuyang Clinical College of Anhui Medical University and the Fifth People’s Hospital of Fuyang City between March 1984 and September 2010. Written consent was provided by all patients undergoing surgical treatment.

All patients had initial symptoms of cyclic abdominal pain during adolescence. The clinical diagnoses of congenital cervical atresia were based on symptoms and abdominal ultrasound before surgery. Diagnoses based on intraoperative exploration were identical to preoperative clinical diagnoses and ultrasound results.

### Examination of uterine tissues

Specimens collected by hysterectomy and biopsy were examined, and their characteristics were recorded, to highlight anatomical characteristics.

### Follow-up

Patient follow-up was conducted for 6 months to 14 years post-operatively. Each follow-up examination assessed cervical presence and dimensions, vaginal dimensions, reproductive outcomes, sexual satisfaction, dysmenorrhea occurrence, and associated abnormalities due to scarring, infection, or other pathologies.

## Results

### Clinical characteristics

**Figure 1:**
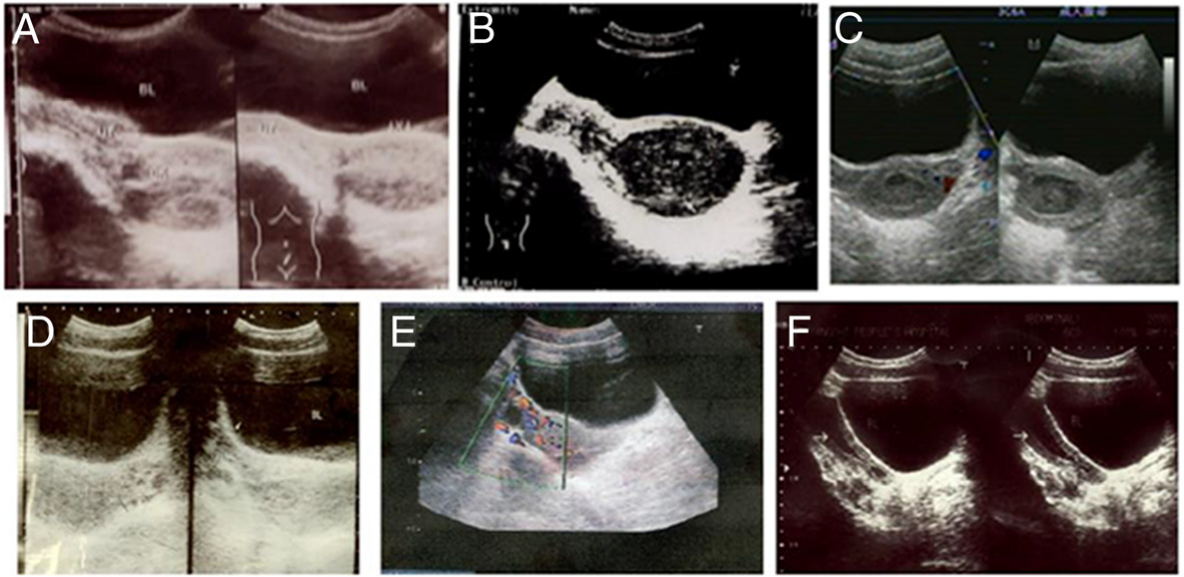
**Characteristics of congenital cervical atresia in preoperative ultrasonography. (A, B)** Type I congenital cervical atresia, showing the unique shape of a Type 1 uterus and uterine isthmus. **(C)** Type II congenital cervical, atresia showing a hematocele in the uterine cavity and a thickened lower uterine cavity (blind end) and wall. **(D)** Type III congenital cervical atresia, showing a sinus-like cervix becoming narrower from the top to the bottom; the end of the cervix was significantly enlarged. **(E)** Type III congenital cervical atresia, showing a long and solid cervix, with no enlarged end. **(F)** Type IV congenital cervical atresia, showing thickening of the wall of the cervical canal and a blind end. The liquid anechoic area of the uterine cavity was bigger than that of the cervix.

**Figure 2:**
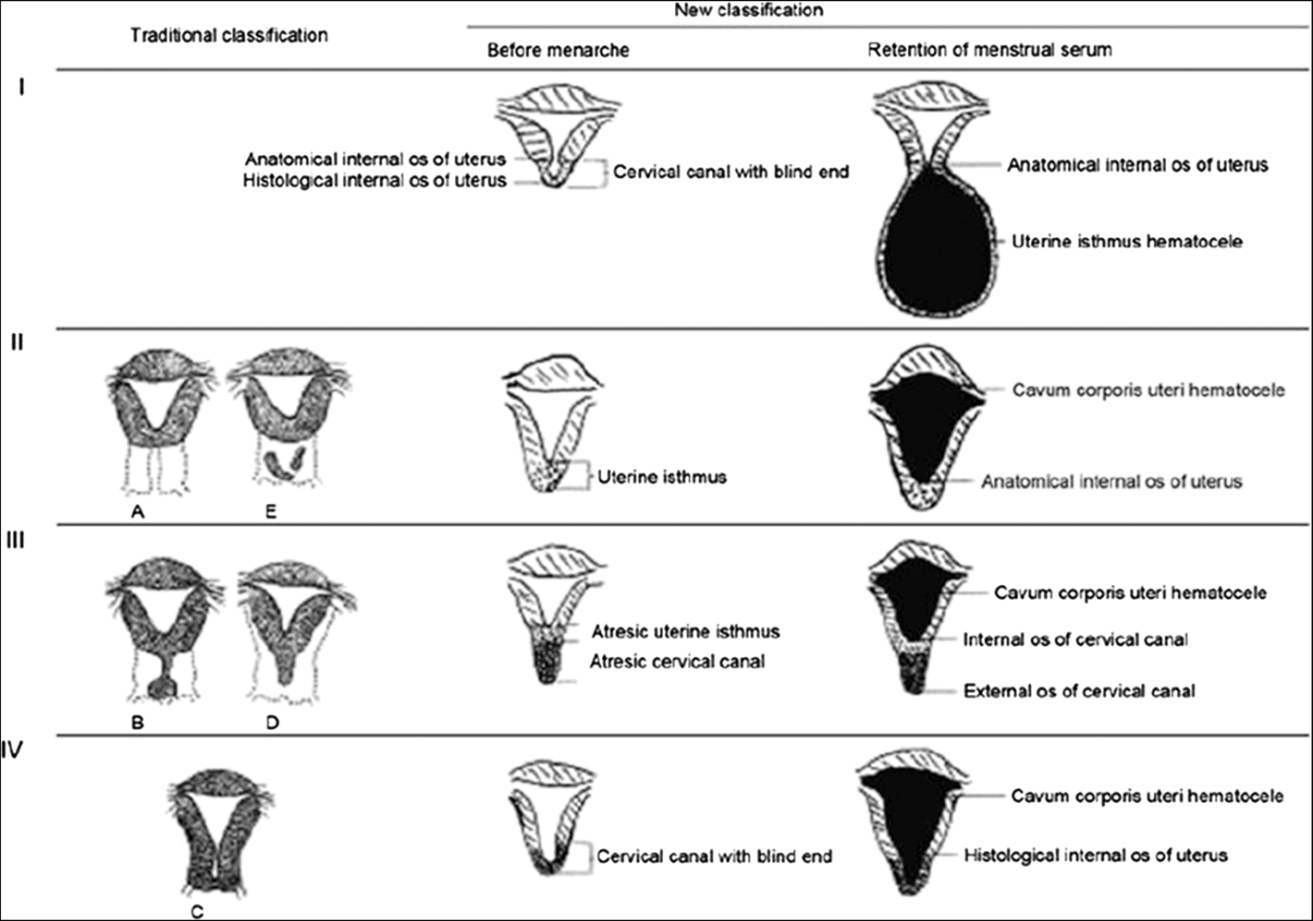
Diagrammatic anatomical comparison between the traditional cervix classification for abnormal development and the proposed cervical atresia typing (coronal uterus view).

**Figure 3:**
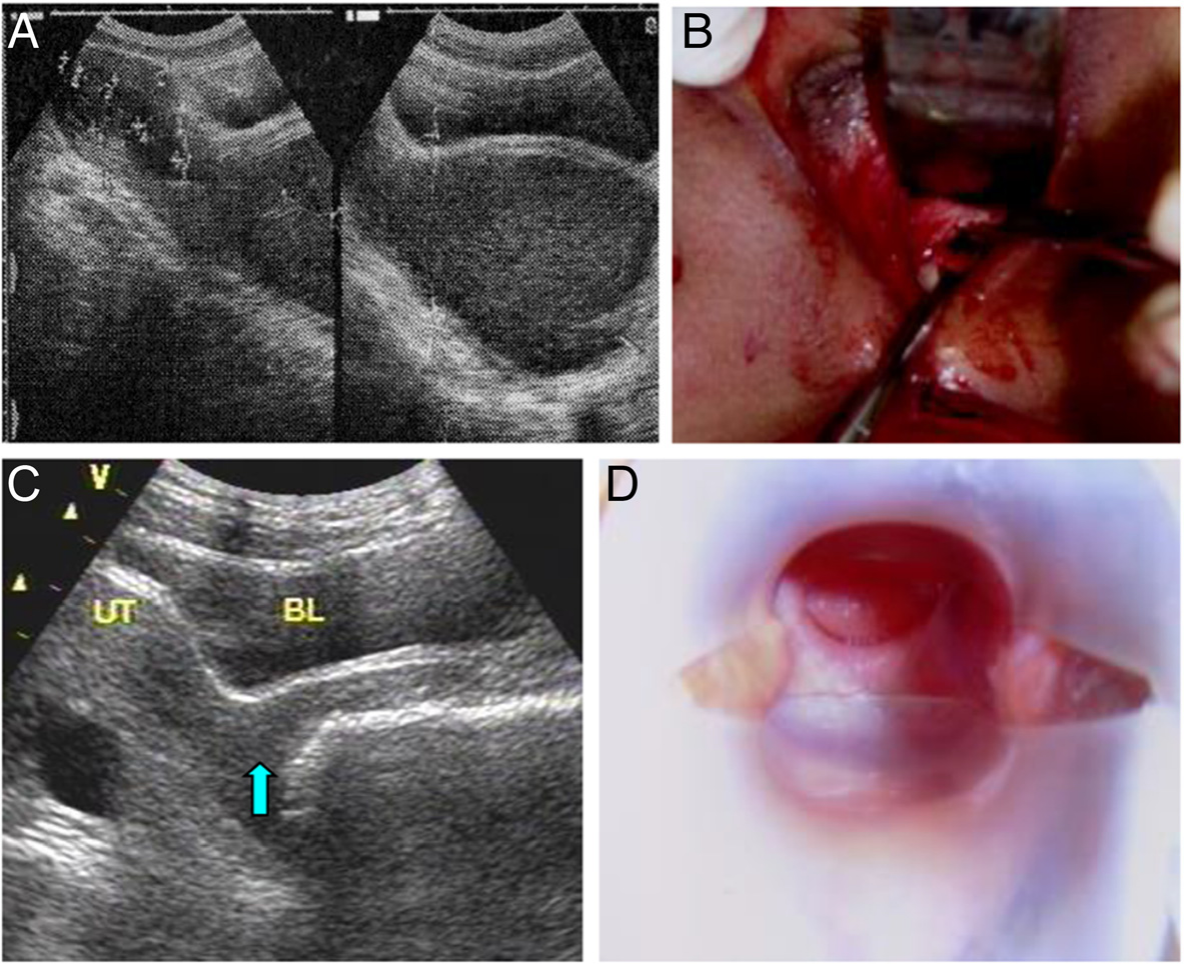
**Type I cervical atresia before, during, and after tracheloplasty. (A)** Ultrasonic image of Type I congenital cervical atresia. **(B)** After the hematoma was discharged, the uterine isthmus cysts became smaller, and the wall consisted of 5 to 6 mm of muscle tissue. **(C)** Fifteen days after the operation, the volume of the uterine isthmus measured 2.1 cm?×?1.5 cm. The arrow in the figure points to the vagina outside the cervix. **(D)** Long-term follow-up showed that the cervix resembled that of a normal nulliparous woman.

**Figure 4:**
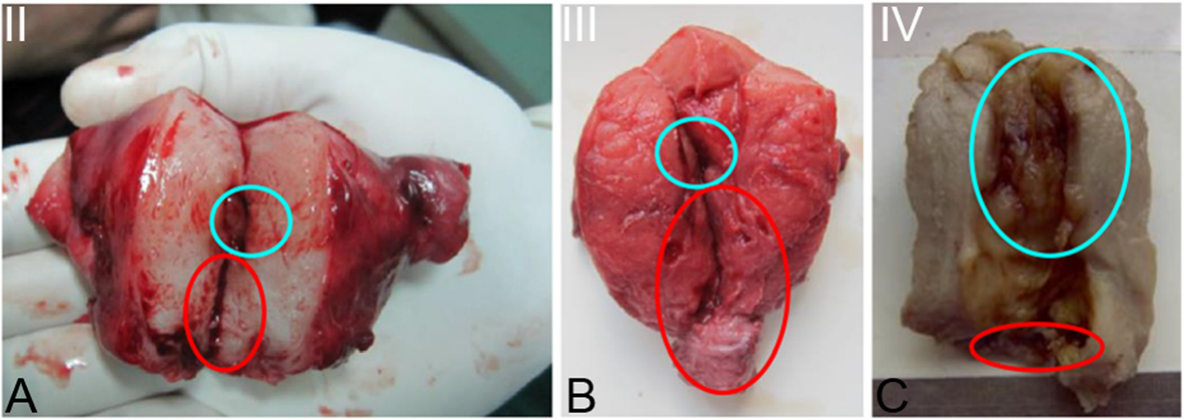
**Sagittal section of the resected uterus of patients with Types II(A), III(B), and IV(C) cervical atresia.** Blue circle indicates uterine cavity, red circle indicates uterine cervix.

A total of 32 patients aged 12 to 21 years with congenital cervical atresia were included in this study. Based on preoperative ultrasound (Figure 1), medical examination, intraoperative exploration, and anatomical and pathological specimen examination, the cases were divided into four types (Figure 2). In each case, ultrasound revealed a liquid accumulation in the uterine cavity that was significantly larger than the cervix. A total of 22 cases (68.8%) were classified as Type I cervical atresia, in which a unique hematoma cyst cavity was detected at the lower portion of the uterus (Figure 1A,B; Figure 3A). Two patients had a normal vaginal recess but with an atresic uterine cervix and a malformed uterine body. In the other 20 cases, no vagina was apparent. Five cases were classified as Type II cervical atresia (5/32, 15.6%), in which a short and solid cervix with a round end was detected (Figure 1C); however, this structure lacked uterosacral and cardinal ligament attachments to the lower uterine body. Two cases were classified as Type III cervical atresia (2/32, 6.3%), in which the lowest region of the uterus exhibited a long and solid cervix (Figure 1D,E). Three cases were classified as Type IV cervical atresia (3/32, 9.4%), in which no internal os was detected, and a blind lumen was found under the uterus (Figure 1F, Figure 2). Sagittal sections of the resected uterus of patients with Types II, III, and IV cervical atresia are shown as Figure 4.

### Surgical treatment and outcome for cases with Type I congenital cervical atresia

Of the 22 patients with Type I cervical atresia, two patients with a blind vaginal canal underwent laparotomy and transvaginal top cervical plastic surgery, while a further 18 patients underwent vaginal and cervical reconstructive surgery, one followed by exploratory laparotomy and one with hysterectomy (owing to an infection sustained after surgery outside our hospital).

In Type I cases, follow-up revealed no dysmenorrhea within 6 months after surgery. In the 20 cases of complete vaginal atresia, two demonstrated vaginal stenosis after removing the vaginal model 4 months after surgery. One patient experienced dysmenorrhea and discomfort during sexual activity 7 years after surgery. A vaginal stenosis ring of 1.5 cm length was detected at the upper vagina, and the collar showed atresia due to scar hyperplasia; therefore, restorative surgery was performed. In one case, the vagina was too shallow, and in 16 cases, the vaginal canal was 8 to 10 cm deep and two fingers wide. Seven of ten patients were married within 3 to 13 years of surgery, and all reported satisfaction with their sex lives. Among these, four patients (4/7, 57.1%) experienced a natural pregnancy, of whom three delivered by lower uterine segment Cesarean section, and one experienced abortion at 8 weeks’ gestational age. Two patients with top vaginal atresia suffered from uterine malformation (complete uterus bicornis and double uterus). The patient with two uteri had one atresic cervix and experienced a natural pregnancy in the other uterus.

### Surgical treatment and outcome for cases with Type II to IV congenital cervical atresia

Among the five patients with Type II cervical atresia, one patient underwent vaginal and cervical reconstruction via abdominal and perineal surgery. During follow-up, the patient reported that she was satisfied with her sex life. However, her cervix gradually shifted to the left rear of the pubic area, and she continued to experience dysmenorrhea. The other four patients received a hysterectomy and vaginal reconstructive surgery.

Among the patients with Type III cervical atresia, two patients underwent hysterectomy and vaginal reconstructive surgery.

Among the patients with Type IV cervical atresia, two of the three patients underwent vaginal and cervical reconstruction via abdominal and perineal surgery. The depth and width of the vaginal canals were normal, and dysmenorrhea did not occur. One patient was married 5 years after surgery and had a natural pregnancy; however, spontaneous abortion occurred at 10 weeks’ gestational age. One patient with uterus unicornis had a hysterectomy and vaginal reconstructive surgery.

### Pathological examination

We studied hysterectomy specimens of Type I (one case), Type II (four cases), Type III (two cases), and Type IV cervical atresia, and biopsy specimens taken from the wall of the hematoma cyst in Type I (six cases) cervical atresia.

**Figure 5:**
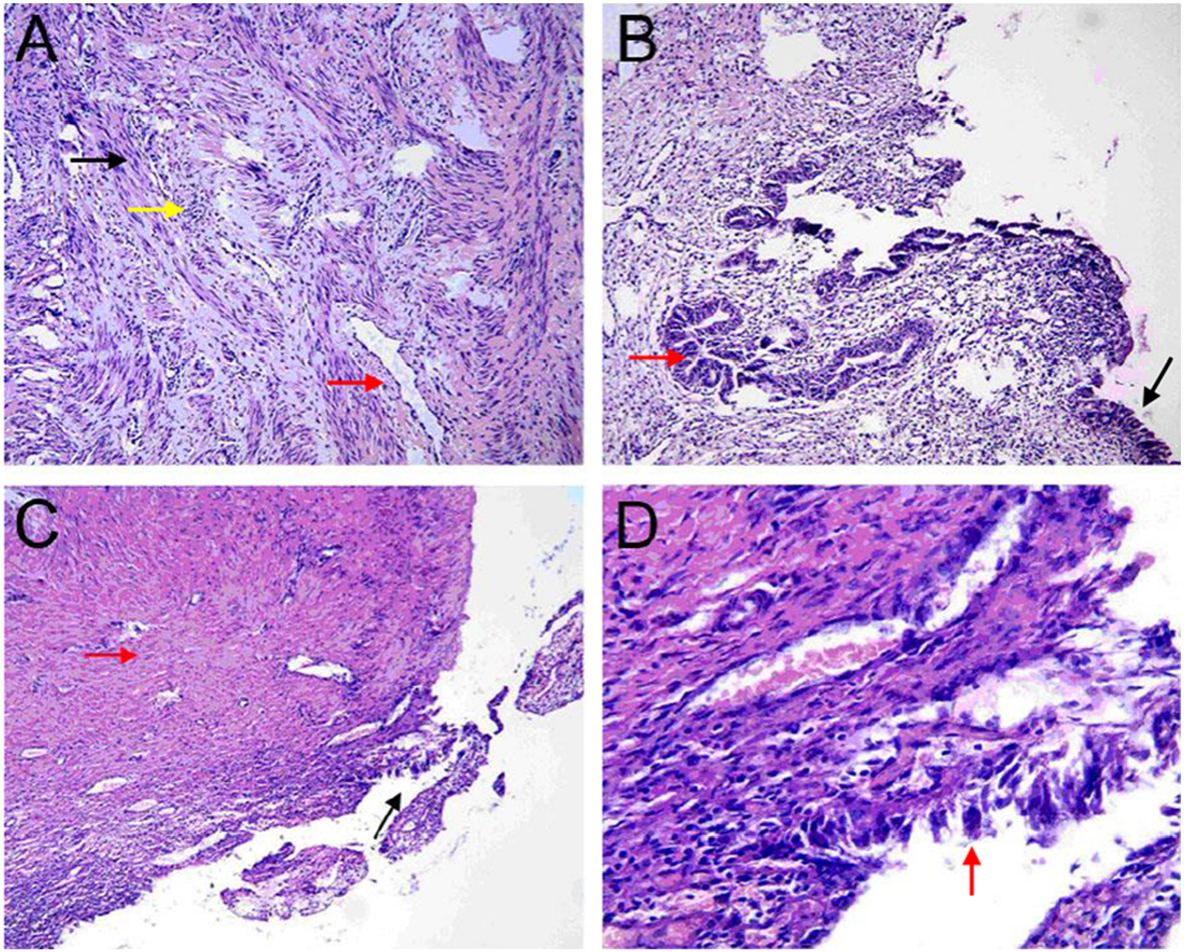
**Type I cervical atresia. (A)** The black arrow indicates smooth muscle. The yellow arrow indicates infiltrated lymphocytes. The red arrow indicates hyperplastic vascular and collagen fiber tissue (HE?×?100). **(B)** The red arrow indicates endometrial glands. The black arrow indicates cervical mucosa (HE?×?100). **(C)** The red arrow indicates collagen fiber tissue. The black arrow indicates cervical mucosal epithelium (HE?×?40). **(D)** The red arrow indicates cervical mucosal epithelium (HE?×?400).

**Figure 6:**
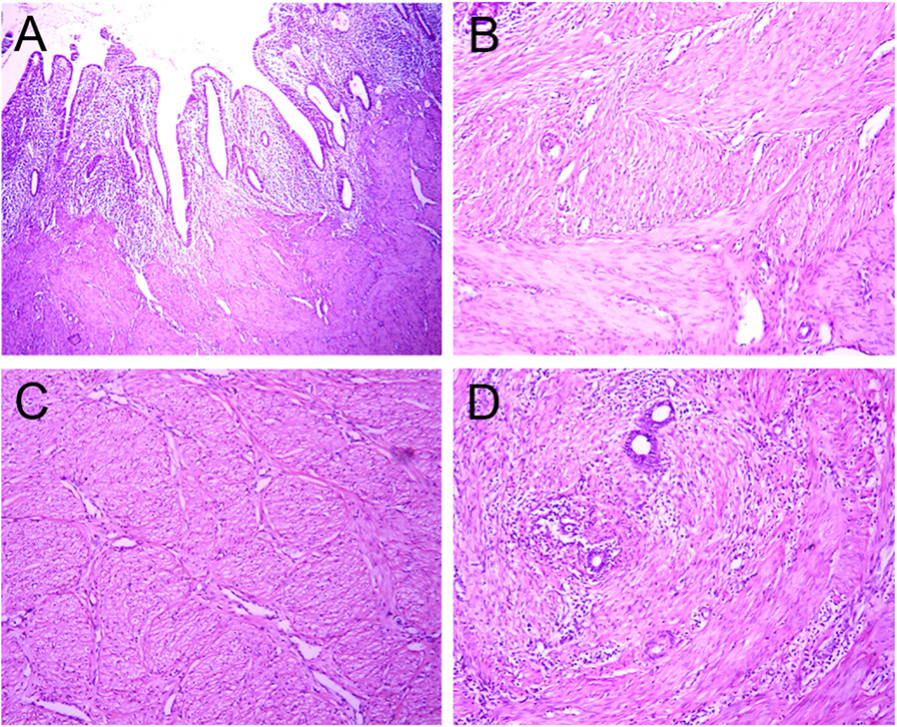
**Type II cervical atresia. (A)** Endometrium and glands on the surface of the uterine cavity (HE?×?40). **(B)** Disorderly and hypogenetic smooth muscle below the uterine cavity (HE?×?40). **(C)** Dysplastic smooth muscle (HE?×?100). **(D)** Muscular layer in the adenomyosis (HE?×?40).

**Figure 7:**
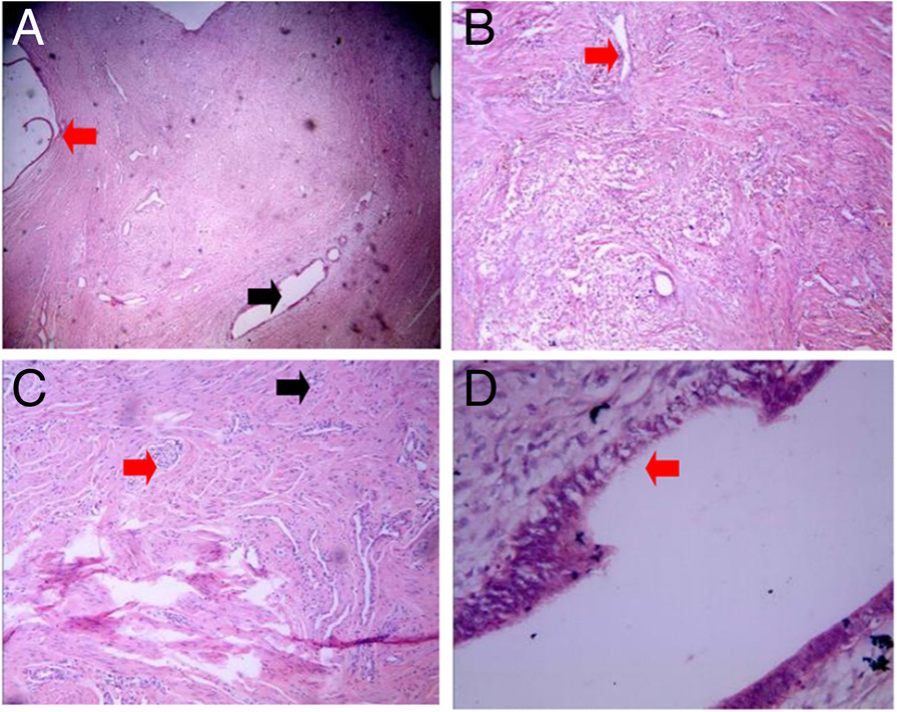
**Type III cervical atresia. (A)** The red arrow indicates cysts in the cervix. The black arrow indicates a lesion of the endometrial glands (HE?×?40). **(B)** The red arrow indicates an old hemorrhage (HE?×?40). **(C)** The red arrow indicates nerve fibers. The black arrow indicates microvessels (HE?×?100). **(D)** The red arrow indicates endometrial glands (HE?×?100).

**Figure 8:**
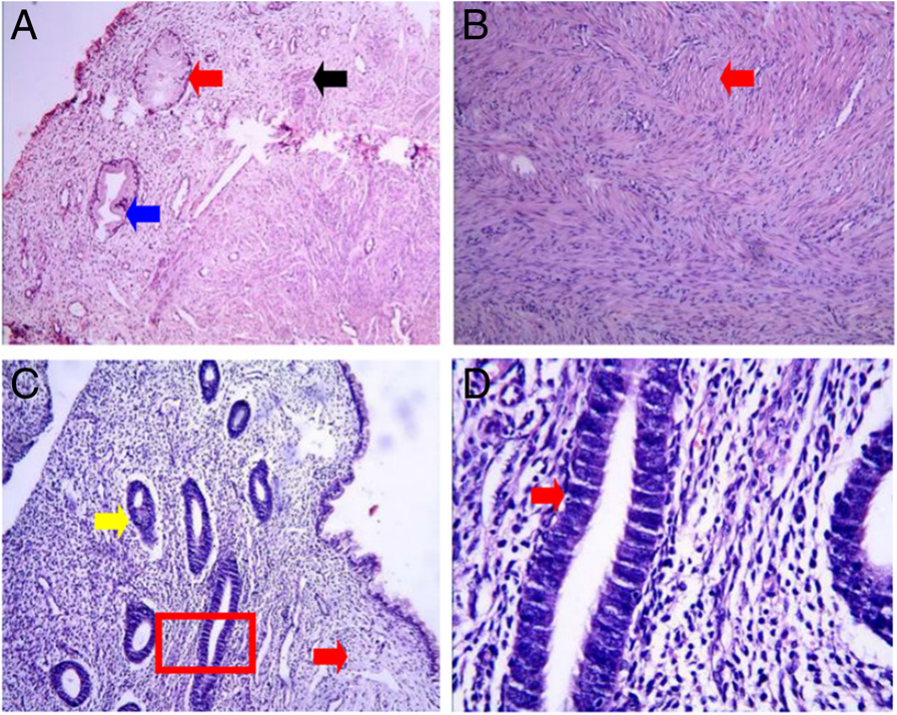
**Type IV cervical atresia. (A)** Cervical canal tissue. Red and blue arrows indicate cervical canal glands. The black arrow indicates a nerve fiber (HE?×?100). **(B)** The red arrow indicates regularly arranged well-developed smooth muscle tissue (HE?×?100). **(C)** Histologicol intemal os of the uterus. The yellow arrow indicates endometrial tissue. The red arrow indicates cervical canal tissue (HE?×?40). **(D)** The red arrow indicates endometrial glands (HE?×?400).

In Type I cervical atresia, the smooth muscle tissues of the hematoma cyst wall are separated by proliferating blood vessels and collagen fibers, with a small amount of lymphocytic infiltration. The cyst cavity surface is coated with cervical mucosa and endometrium (Figure 5). In Type II cervical atresia, the surface of the cavity consists of uterine endometrium and glands, which covers a disorderly hypogenetic smooth muscle layer. In one case, the uterine cavity extended downward, showing a short tubule and visible cervical mucosa. A retention cyst with an old hemorrhage in the atresic cervical tissues was observed (Figure 6). In Type III cervical atresia, the atresic cervix is composed of fibro-muscular tissue, fibrous tissue, and collagenous fibers, with scattered nerve fibers, some smooth muscle bundle, and small blood vessels. In one case, a local retention cyst and a focus of adenomyosis were seen (Figure 7). In Type IV cervical atresia, the uterine endometrium is replaced by cervical mucosa at the junction of the uterine body and cervix. The lower wall of the blind end of the cervical canal is mainly composed of collagenous fibers, with scattered small blood vessels and smooth muscle bundles (Figure 8).

## Discussion and conclusions

Anatomical characteristics of individual cases of congenital cervical atresia should be considered distinct from other Müllerian duct anomalies in terms of diagnosis and treatment selection. Our observations of clinical characteristics during preoperative ultrasound and intraoperative exploration identified four types of congenital cervical atresia. Type I cervical atresia is characterized by a normal functioning anatomic internal os, a relatively large hematoma cyst, and accumulations of blood in the uterine cavity. In Type II cervical atresia, a shortened cervix and detached sacrum and main ligament may resemble Müllerian fusion of the middle and tail sections [17]. Type III cervical atresia is characterized by long and solid tissues below the uterine body, wherein both the uterine isthmus and cervical canal are atresic. In Type IV cervical atresia, the cervix attaches to the normal ligament and connections form between the uterine body and the blind end of the cervical canal. Absence of the uterine isthmus is characteristic of Type IV, resulting in expansion of the uterine cavity and blood accumulation during menstruation.

**Figure 9:**
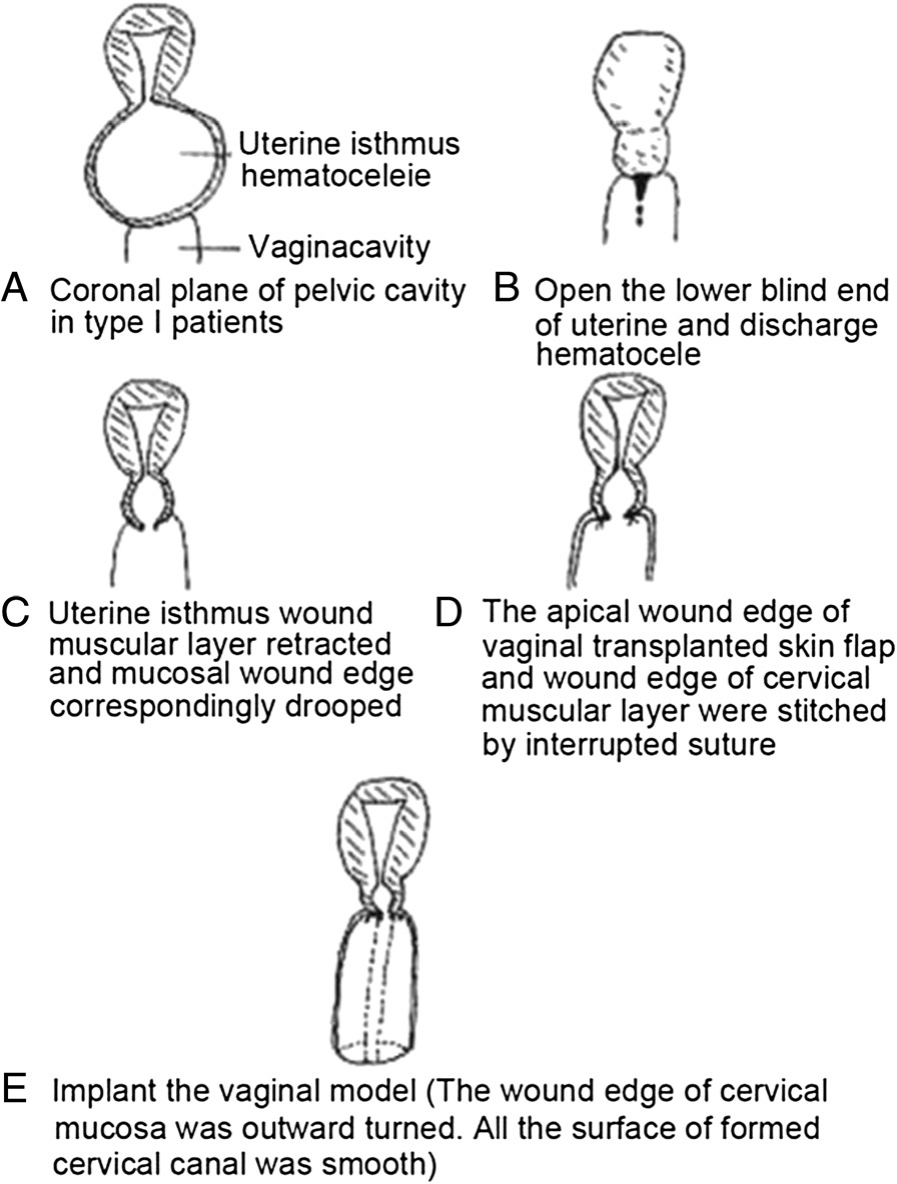
**Vaginoplasty and tracheloplasty performed through a perineal approach in Type I cervical atresia patients. (A)** Coronal plane of pelvic cavity in Type I patients. **(B)** The lower blind end of the uterus is opened and the hematocele is discharged. **(C)** The uterine isthmus wound muscular layer is retracted and the mucosal wound edge is correspondingly drooped. **(D)** The apical wound edge of the vaginal transplanted skin flap and the wound edge of the cervical muscular layer are stitched by interrupted suture. **(E)** The vaginal model is implanted. (The wound edge of the cervical mucosa was outward turned. The surface of the created cervical canal was smooth).

Observations of the anatomical characteristics of cervical atresia can provide a guideline for selection of appropriate surgical techniques. For Type I cervical atresia with a normal uterine body, vaginal and cervical reconstruction was performed with perineal surgery (Figure 9; Figure 3). The uteri of Type I patients who underwent restorative surgery for cervical atresia could be successfully preserved. Type I cases were generally able to retain uterine structures, reduce dysmenorrhea, and had the potential to achieve natural pregnancy. In some patients, the reconstructed cervix formed the lower uterine segment during pregnancy, and the patients underwent successful lower uterine segment Cesarean Section. A total of 60% of Type I patients received appropriate treatment and preserved their physiological and reproductive function. For Type II and III cervical atresia, hysterectomy was performed and vaginoplasty was carried out. For Type IV cervical atresia, the treatment principles were similar to those of Types II and III cervical atresia. The uterus could be preserved if the patients requested. However, as the patients of Type IV cervical atresia had no uterine isthmus, the lower uterine segment during pregnancy could not be formed, and the patients were infertile [18].

While further studies will be required to extend our observations, knowledge of clinical characteristics of congenital cervical atresia obtained during preoperative ultrasound and intraoperative exploration may improve treatment outcomes, including the reduction of recurrent atresia, a decrease in long-term dysmenorrhea, and the retention of reproductive capacity. Additionally, the risk of post-surgical hysterectomy may be reduced. The results from this study should be interpreted in terms of novel treatment approaches for congenital cervical atresia, such as combined retropubic balloon vaginoplasty and laparoscopic canalization and laparoscopic canalization under vaginoscopic monitoring [19,20], which represent minimally invasive management techniques with promising short-term outcomes.

In summary, we explored the anatomic location, ultrasonic features, surgical treatments, and outcomes of 32 cases of congenital cervical atresia. We identified four distinct types of congenital cervical atresia, which provided a guideline for selection of appropriate surgical techniques. Our results may provide insight into the development and use of novel treatment approaches for congenital cervical atresia.

## Competing interests

The authors declare that they have no competing interests.

## Authors’ contributions

ZX: surgery design, cases collection, follow-up, data analysis and manuscript writing; XZ and NZ: operation and follow-up; HX, YL, LL and XL: clinical work; JL: pathological examination. All authors read and approved the final manuscript.
